# Superbat: battery-like supercapacitor utilized by graphene foam and zinc oxide (ZnO) electrodes induced by structural defects

**DOI:** 10.1039/c9na00199a

**Published:** 2019-05-10

**Authors:** Sibel Kasap, Ismet I. Kaya, Sergej Repp, Emre Erdem

**Affiliations:** SUNUM Nanotechnology Research Centre, Sabanci University TR-34956 Istanbul Turkey emreerdem@sabanciuniv.edu; Faculty of Engineering and Natural Sciences, Sabanci University TR-34956 Istanbul Turkey; Institute of Physical Chemistry, University of Freiburg Albert str. 21 79104 Freiburg Germany

## Abstract

The current work presents a hybrid type of energy storage device composed of both graphene foam and zinc oxide electrodes, which exhibits both the electrochemical performance of a supercapacitor with a relatively higher power density, and a battery with a relatively higher energy density as compared to each individual component as single devices. Te hybrid's improved performance was correlated to the defective structure of the electrodes. To enhance the electrochemical performance of supercapacitors, it is necessary to have a well-defined mass, shape, and surface area of electrode materials. Here, we present an original design of a mounting device that enabled precisely determining all the critical parameters of electrode materials for a particular mass and surface area. With the aid of our original setup, we produced a supercapacitor device that could also act as a battery due to its high energy density values, hence we named it as *superbat*. In this work, 3D graphene foam was used as the first electrode due to its large surface, while for the second electrode, ZnO nanocrystals were used due its defective structure. Paramagnetic resonance Raman and impedance spectroscopy were performed in order to understand the origin of the performance of the hybrid capacitor in more depth. In particular, we obtained a high specific capacitance value (*C* = 448 F g^−1^), which was exceptionally related not only to the quality of the synthesis but also the choice of electrode and electrolyte materials. Moreover, each component used in the construction of the hybrid supercapacitor also played a key role in to achieving high capacitance value. The results demonstrated the remarkable performance and stability of the *superbat*.

## Abstract 2

The current work presents a hybrid type of energy storage device composed of both graphene foam and zinc oxide electrodes, which exhibits both the electrochemical performance of a supercapacitor with a relatively higher power density, and a battery with a relatively higher energy density as compared to each individual component as single devices.

## Graphical abstract

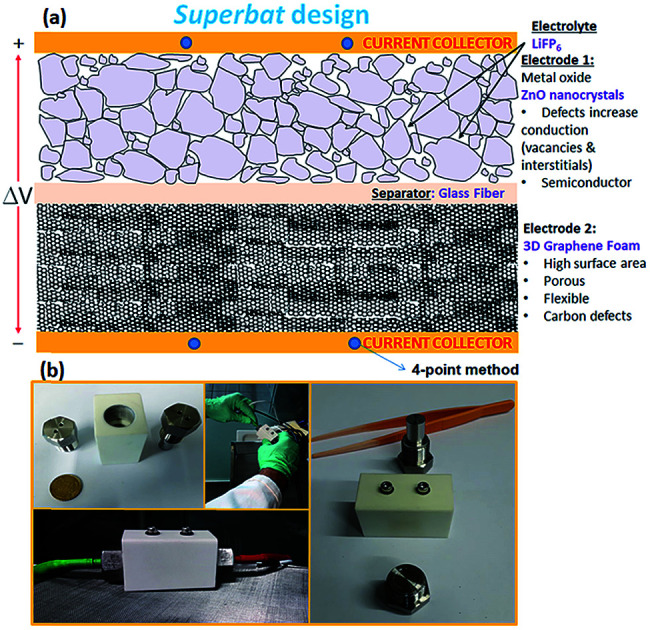

## 1. Introduction

Exploring the properties of materials for battery and supercapacitor applications is one of the “dirtiest” kinds of research, not only because of their hazardous basic components, such as electrodes, electrolytes, and their solvents, separators, and current collectors but also the need to go through the thousands of publications in the literature, which present an enormous number of electrochemical performance results. Electrode materials are often used in supercapacitors or batteries with little or no investigation of their defect structures, which in fact play a crucial role in their electronic, magnetic, optical, and mechanical behaviors, and thus, in this context, their electrochemical performance. Electrodes are key components in the production of supercapacitors and the strategies for selecting the right electrodes play a decisive role in their specific power and energy storage performance. For instance, one strategy is to decrease the crystal size to the nanometer scale in order to increase the surface area and to have more active sites and shorter ionic paths. Another strategy is to use composite materials to achieve a higher conductivity and more structural stability. Such critical decisions in the selection of electrode materials make supercapacitor applications highly diverse and open to new developments. Today, batteries and supercapacitors are introduced as the principal energy storage media for so-called clean-energy or green-energy in electrically driven devices, such as electric vehicles. Lithium, some transition metal ions, such as Co, Mn, and Fe, and carbonaceous materials, including graphite, graphene oxide, and carbon black, are the most common electrode materials in batteries, whereas lithium hexafluorophosphate (LiPF_6_) is the most common electrolyte salt. Therefore, when speaking about “clean-energy” in electricity production and storage, the hazards of such materials also need to be taken into serious consideration. However, such environmental issues, which may play a role in energy policies, are not the main focus of this work but the defect structures are.

Thanks to the tremendous progress in the research and technology in the energy field, supercapacitor-based electrical energy storage devices have recently become one-step closer to being a viable alternative to the traditional Li-ion batteries due to the enhancements made in their energy storage capacity.^1–3^ Indeed, supercapacitor technology could reach to a milestone, where energy storage systems can both have a high energy capacity and deliver high output power by combining multifunctional materials with smart designs. On the other hand, our recent investigations on supercapacitors^4,5^ motivated us to tackle some challenges that still need to be addressed for building better supercapacitors. There are different types of supercapacitors in terms of their operating mode principles and designs, namely electrostatic double-layer capacitors (EDLCs), electrochemical pseudocapacitors, and hybrid capacitors. It is well known that pseudocapacitors are capable of storing much more electrical energy compared to standard supercapacitors. In this work, electrochemical pseudocapacitors were used with a distinct efficient design, where the supercapacitor may act as a battery. Such a system can be called a “*superbat*” and may have potential applications in future electric vehicles, wearable electronics, nanogenerators, and microelectronic devices. In fact, *superbat* construction is similar to that of batteries but the principle of operation is different. In a *superbat*, there are two electrodes, a separator, and an electrolyte containing negative and positive ions, as in a battery. In the charging state, negative and positive ions move toward the electrodes and they are absorbed on their surface. Such reactions occur very fast and hence enable a *superbat* to deliver high power. To reach the ultimate goal of high energy storage, the rated potential of a *superbat* needs to be improved, simply because of the fundamental energy density relation: 
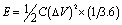
, where *E* is the stored energy density (W h kg^−1^), Δ*V* is the operating voltage (*V*), and *C* is the specific capacitance (m^−2^ kg^−2^ s^4^ A^2^ that is generally given in cgs units as: F g^−1^). Consequently, the maximum power density of a *superbat P*_max_ can be determined from the low frequency result of impedance *via* the relation: *P*_max_ = *V*_max_^2^/4*m*_t_*R*_ESR_, where *V*_max_ is the maximum charging voltage, *R*_ESR_ is the equivalent series resistance, and *m*_t_ is the total mass (kg) of both electrodes. *P* and *E* are related to each other through time as follows: *P* = 3600(*E*/Δ*t*), where Δ*t* is the discharge time (s). Obviously, in this perspective one has to ensure the stability of the electrolyte, which can be degraded beyond a critical potential, decomposed into a gas state, and can then become unstable (*i.e.*, 4.2 V for LiPF_6_). To understand the electrochemical performance of the *superbat* systems, we first studied the defect structures of the electrode materials that were used in this work, namely graphene foam (GF) and ZnO nanocrystals, by electron paramagnetic resonance (EPR) spectroscopy. EPR is well suited to aiding understanding the role of structural defect centers since it provides a direct method to monitor the different paramagnetic states of defects. Therefore, it complements other analytical experimental techniques, such as Raman spectroscopy. Moreover, due to the insufficient analytical characterization of electrode materials and the lack of data from advanced EPR techniques, which preclude the unambiguous determination of defect states, the role of structural point defects on electrochemical performance remain unclear and is still a matter of debate. This is mainly due to the fact that, at present, most researchers who investigate supercapacitors or batteries are trying to improve the energy or power density, charge/discharge time, and/or cycling stability, while typically overlooking the effect of defect structures at the microscopic scale. For instance, one of the rare defect studies in the literature unambiguously demonstrated that structural defects in the crystals of NaCrO_2_ electrodes play a significant role in achieving a high specific capacity and excellent capacity retention for sodium ion batteries.^6^ In another study, which was about the critical role of point defects in δ-MnO_2_ nanosheets, it was shown that Mn vacancies provide ion intercalation sites, which simultaneously enhance the specific capacitance, charge-transfer resistance, and cycling stability.^7^ Nowadays, due to their high specific capacitance and low resistance, metal oxides, such as ZnO, In_2_O_3_, MnO_2_, and RuO_2_, have great potential in designing superbats with high energy and high power density.^8,9^ Very recently, it was reported that ZnO-based composite electrode materials can provide good electrochemical reversibility, high specific capacitance, high power density, high energy density, and good cycling stability, which makes them promising materials for advanced supercapacitors.^10^ In particular, ZnO is a highly defective wide band-gap semiconductor and a luminescent material, in which the defect centers play a vital role in its ionic and electronic transport properties. Conceivably, some of the possible intrinsic defect centers in ZnO are: (i) zinc vacancies, (ii) zinc on interstitial sites, (iii) oxygen on interstitial sites, and (iv) oxygen vacancies. EPR-active intrinsic defect centers have previously been discussed in detail elsewhere.^11–14^ Within the framework of our recent EPR studies on ZnO nanoparticles, two distinct EPR spectral features were identified as defect centers, located at the core and on the surface with *g*-factors of 1.962 and 2.004, respectively.^14–16^ The difficult task of understanding the origin of intrinsic defects and their assignment, both at the bulk and at the surface of ZnO nanocrystals synthesized *via* various routes can be partly solved by the semi-empirical core–shell model. EPR and photoluminescence spectroscopic investigations have been instrumental in explaining the origin of different defect types in nanosized ZnO as well as the temperature dependence and microwave saturation behavior of localized and delocalized electrons by that model.^11,12,17^ On the other hand, carbonaceous materials are also highly defective; where defects such as dangling bonds and C-radicals can be detected *via* the EPR technique.^18,19^ Recent EPR studies delivered detailed information on the defect structure of carbonaceous materials, not only providing an insight into their spin properties, which includes the conduction electrons, unpaired spins, and dangling bonds, but also enabling an investigation of the electronic states in the different forms of carbon. Even ZnO decorated with hybrid graphene materials has revealed interesting EPR results, indicating competing and healing effects of the intrinsic defects involved both in ZnO and graphene.^19^

Graphene-based composites, in particular, have recently been attracting lots of attention, due to their potential application in energy storage devices, such as batteries and supercapacitors.^1,20^ Graphene possesses a single atomic layer, where sp^2^ hybridized carbon atoms are arranged in a honeycomb-like two-dimensional (2D) network. Such an exclusive structural peculiarity results in a tunable large specific surface area and high thermal conductivity as well as a very high intrinsic mobility of charge carriers.^21^ However, 2D graphene sheets have a strong tendency to aggregate and to restack in the macroscopic scale because of the strong π–π interactions between individual sheets. This tendency leads to a reduction in the effective surface area and limits the surface available for electrochemical double-layer formation. Therefore, the usage of 2D graphene sheets in electrochemical applications is limited.^22,23^ To overcome these drawbacks, three-dimensional (3D) macroscopic graphene structures, including sponges, foams, hydrogels, and aerogels, have been developed, recently.^24^ These macroscopic structures not only retain most of the precious properties of 2D graphene, such as a high electrical conductivity and large specific surface area, but also offer low density and good mechanical strength.^25–28^ Among these, foam-like 3D graphene structures have attracted lots of attention in electrochemical applications because of their porous and well-defined morphology. The GF morphology not only ensures fast electron transfer but also allows combining other electrochemically active materials, which can enhance the supercapacitive performance.^22,27^ The utilization of a self-assembly method to prepare graphene oxide (GO) followed by a reduction process using strong chemical oxidants is the most common method to produce foam-like macroscopic structures. Although a massive amount of 3D-GF can be produced by this technique, foam structures contain defects and exhibit low electrical conductivity.^29–31^ Contrary to the self-assembly method, the chemical vapor deposition (CVD) method can produce 3D graphene foams with more controlled and uniform morphologies and structures with a large surface area. *Via* this process flow, free-standing graphene foams with high electrical conductivity and improved structural stability superior to the chemically derived graphene sheets can be produced.^32,33^ From an electrochemical performance point of view, several graphene-decorated models have already been introduced to enhance the capacitance for energy-storage applications. Such models have shown a significant improvement in capacitance, attributed to extra and deeper redox reactions, due to the enhancement of conductivity *via* the defect structures and dispersion due to the incorporation of graphene.^34,35^ Therefore, in any energy-storage device, such as a battery or a capacitor, the role of defect structures in the electrode materials needs to be thoroughly investigated. Recently, the importance of vacancies in functional materials for clean energy storage and harvesting was reported as “the perfect imperfection”, whereby by extensively controlling the defect centers, researchers can influence the materials physical and chemical properties, such as the band gap and conductivity, and thus the electrochemical performance of electrode materials.^36^

The basics of the above-mentioned properties and applications of carbonaceous materials have already been presented in a number of excellent comprehensive and focused reviews or monographs under the framework of supercapacitor technology.^9,37–40^

However, there have been only a few EPR studies performed on graphene and graphene-like structures, such as 3D-GF. For example, two distinct EPR spectra of reduced graphene oxide (rGO) were reported: a broad spectrum at *g* = 2.0027, which could be attributed to graphite-like carbon, and a narrower spectrum at *g* = 2.0028, associated with carbon radicals. Since graphene is also phonon-mediated active carbon, the electron–phonon interaction plays an important role in the hybridization mechanism, which is essentially responsible for the large surface area. However, a number of unclear issues, such as the Kohn anomaly, the broadening/shift in D- and G-bands, the anharmonicity, and multi-phonon contributions, may be closely related with the defects. The importance of phonon–defect interactions as an alternative cause for the anomalies of graphene has been reported in previous reports. Therefore, a correlation of the Raman and EPR spectroscopy results could conceivably give information about the defect-related electron–phonon or phonon–defect interactions, which most likely have an effect on the electrochemical performance of energy storage and power devices, and hence *superbat*s. Both of these versatile experimental methods complement each other in exploring the fundamental properties of graphene and ZnO and for characterizing the type of defects, defect–phonon interactions, and electrical transport.^5,41,42^

In the present study, basically, a hybrid superbat was assembled from four main components: graphene foam and ZnO nanocrystals as next generation electrodes, 1 M LiPF_6_ in ethylene carbonate and ethyl methyl carbonate (EC/MC = 50/50) as the electrolyte, and glass fiber as the separator. Overall, the reason why we used these materials and our main strategy to increase the device performance is as follows: unlike EDLCs, pseudocapacitors store energy through Faradaic reactions, comprising fast and reversible redox reactions between electrode materials and an electrolyte on the electrode surface. Usually, pseudocapacitors containing metal oxides reveal higher capacitance than those of EDLCs due to their different charge-storage mechanism. However, they often suffer from stability problems due to their low electrical conductivity. The hybrid superbat is a smart arrangement of EDLCs and pseudocapacitors, in which EDLCs and the Faradaic capacitance may contribute to the overall performance instantaneously. Moreover, the existence of defect structures in ZnO and GF and their synergetic effects on the hybrid *superbat* could improve the overall conductivity of the system, and thus the electrochemical stability and the performance. This is quite reasonable, while the high conductivity can contribute to the reduction of internal resistance, resulting in a high power density. However, on the one hand, it is well known that defects can improve the conductivity of ZnO, while on other hand, as the defects increase in a metal/semi-metal, such as a graphene, the conductivity could be expected to decrease. So it is quite a challenging issue to understand the mechanism of how such intrinsic defects can affect the performance of a complete supercapacitor device. It is clear that competing effects will occur and such effects will mainly affect the performance of the device. Thus it is quite difficult to say how the defects of GF and ZnO influence individually the performance of the *superbat*. Nevertheless, equipped with such supreme properties, *superbat*s are starting to emerge as an efficient energy storage technology that may play an essential role in the energy systems in the future.

## 2. Experimental

### (A) Synthesis of ZnO nanocrystals

In order to understand the effect of the defects, we used a specific method to synthesize defective ZnO nanocrystals. A solid-state (SS) synthesis method enabled us not only to characterize the defect centers under different conditions but also allowed for a good control of the mean size, size distribution, and morphology of the nanocrystals. A detailed description and characterization of the SS-synthesized ZnO nanocrystals can be found elsewhere.^13^ Briefly, in this method, we used the following chemicals with the highest purity available: zinc sulfate (ZnSO_4_, Aldrich, purity 99%) and sodium hydroxide (NaOH, Roth, purity 99%). We ground NaOH and ZnSO_4_ with a molar ratio of 2 : 1, respectively, in a mortar for 30 min at room temperature (RT). After that, we washed the solid mud by adding Millipore water and then washed the suspension several times. Finally, the powder was dried for 2 h at 80 °C in an oven. We obtained ZnO nanocrystals by calcinating this precursor at different calcination temperatures (CT) ranging from 300 °C up to 600 °C. The calcination process was done as follows: 23 °C (RT) (heating rate 8.3 °C min^−1^) → CT (2 h) → free cooling to RT. The product was ground with a pestle and mortar. The chemical equation in the SS reaction is as follows:

1 ZnSO_4_ + 2NaOH → ZnO + Na_2_SO_4_ + H_2_O

### (B). Synthesis of graphene foam

The 3D-GF macrostructure was synthesized on nickel foam with 1.6 mm thickness (≥95% porosity, 99.99% purity) *via* a chemical vapor deposition (CVD) method. The nickel foam (5 × 5 cm^2^) was placed horizontally in the central zone of the quartz tube in the CVD chamber and heated up to 1000 °C with a flow of Ar (250 sccm) and H_2_ (100 sccm) gas mixture at low pressure (60 mbar). To remove the oxide layer on its surface, the nickel foam was maintained for 10 min under that condition. Then, 100 sccm CH_4_ gas as a carbon precursor was delivered into the quartz tube for 60 min to grow graphene sheets on the nickel template. Following this step, the sample was allowed to cool to ambient temperature naturally under an Ar gas flow. In order to obtain the free-standing graphene macrostructure, the nickel template was etched in 1 M FeCl_3_ solution for 24 h. After the etching, the 3D graphene foam was immersed in a DI-water/HCl mixed solution (1 : 1 (v/v)) to clean it of iron residues. Finally, the graphene foam was dried at 80 °C for 30 min in an oven.

### (C). Design of the *superbat*

**Fig. 1:**
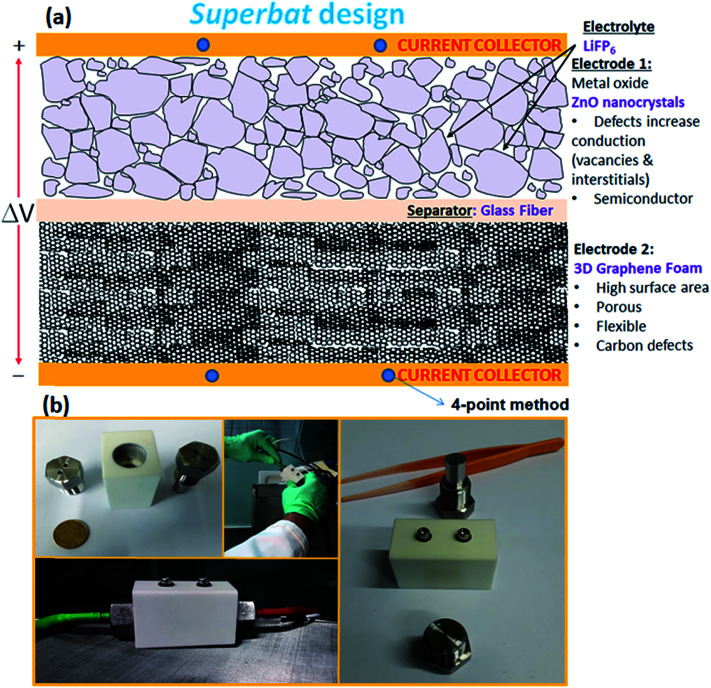
(a) The *superbat* design consisting of four basic components. Advanced functional electrodes: 3D graphene foam and ZnO nanocrystals, separator: glass fiber, and electrolyte: 1 M LiPF6 in EC/MC = 50/50. (b) The homemade capacitor mounting system.

The design sketch and all of the components of the *superbat* can be seen in the illustration in Fig. 1(a). The photographs in Fig. 1(b) present the prototype homemade capacitor mounting system. The main difference in the *superbat* design compared to a conventional two-electrode setup, such as the commercial T-shape Swagelok two-electrode system, is the defined diameter and weight of the electrodes and also the good control of the insertion of them into the device. Moreover, stainless steel current collectors are highly effectively designed to measure the current and voltage. This device practically did not require measuring the electrochemical response of both electrodes independently in a three-electrode device to study the reactions taking place, which was useful as here the main aim was to see directly the synergy of the materials all together.

### (D). Methods

Scanning electron microscopy (SEM) (JEOL, JSM-6010LV) and Raman spectroscopy (Renishaw inVia, excited by a 532 nm laser) were used to characterize the 3D graphene foam. The structure of the 3D foam-like graphene was verified by Raman data, which were recorded under a 532 nm laser source under a 2 μm spot size. The 3D structure of graphene foam was analyzed by SEM imaging at 10 kV. X-band (9.86 GHz) EPR measurements were performed with a Bruker EMX spectrometer using a rectangular TE102 (X-band) resonator. The magnetic field was determined using an NMR gaussmeter (ER 035M, Bruker); for calibration of the magnetic-field measurement, a polycrystalline DPPH with *g* = 2.0036 was used. Cyclic voltammetry (CV), electrochemical galvanostatic cycling with potential limitation (GCPL), and electrical impedance spectroscopy (EIS) measurements were carried out using a VSP/VMP3 multichannel potentiostat/galvanostat (BioLogic) at room temperature. The CV tests were conducted at a scan rate of 10 mV s^−1^. The GCPL tests were carried out at a rate performance between 0.1 and 5C. Electrochemical analysis, in particular the specific capacity, was calculated from the GCPL data with the aid of EC-LAB V10.40 *BioLogic* software. EIS data were collected in the frequency range between 0.1 Hz and 100 kHz. For the analysis of the spectra, we used *ZView* software. The amplitude of voltage modulation was set to 100 mV. A four-point electrode-setup was used to exclude the potential drop due to electrochemical reactions occurring at the working electrode(s). The setup consisted of steel screws as the current collector, on which the electrode material was connected. Both electrodes were separated by a glass fiber and 1 M LiPF_6_ in EC/MC = 50/50 was used as the electrolyte.

## 3. Results and discussion

**Fig. 2:**
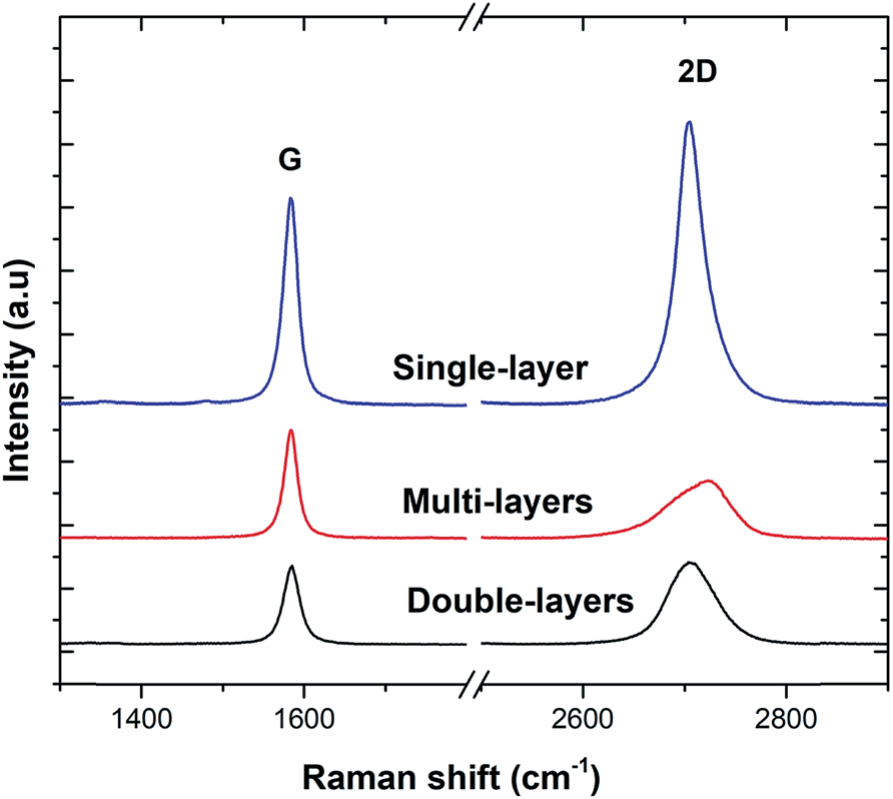
Raman spectra of 3D-GF obtained at different positions.

Three sample Raman spectra taken at different points on the 3D-GF are shown in Fig. 2. Graphene specific G and 2D peaks at 1590 cm^−1^ and 2700 cm^−1^, respectively, can be seen clearly in these spectra. The ratio of the signal intensities at the 2D and G peaks confirmed that 3D-GF had a varying thickness, and in the sample traces, single-, double-, and multi-layer graphene were identified. In addition, the absence of the D-band around 1350 cm^−1^, an indication of defects and disordered carbon, signified that the CVD-grown 3D-GF was of high quality.^43^

**Fig. 3:**
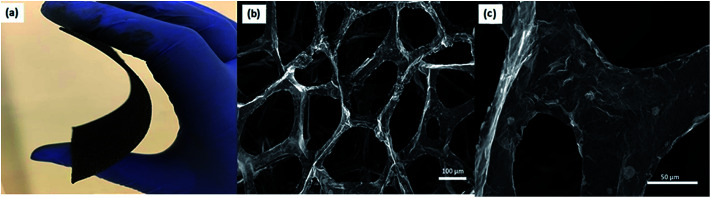
(a) Photograph of 3D-GF, (b and c) SEM images of the 3D-GF with different magnifications.

Fig. 3(a) shows a photograph of the as-prepared GF, which exhibited a monolithic form with good mechanical stability, and hence, it could be easily handled. Fig. 3(b) and (c) show the SEM images of 3D-GF at different magnifications. As revealed by the photographs, a smooth graphene skeleton with a 3D structure was maintained without any cracks, validating the interconnected network of the graphene foam.

**Fig. 4:**
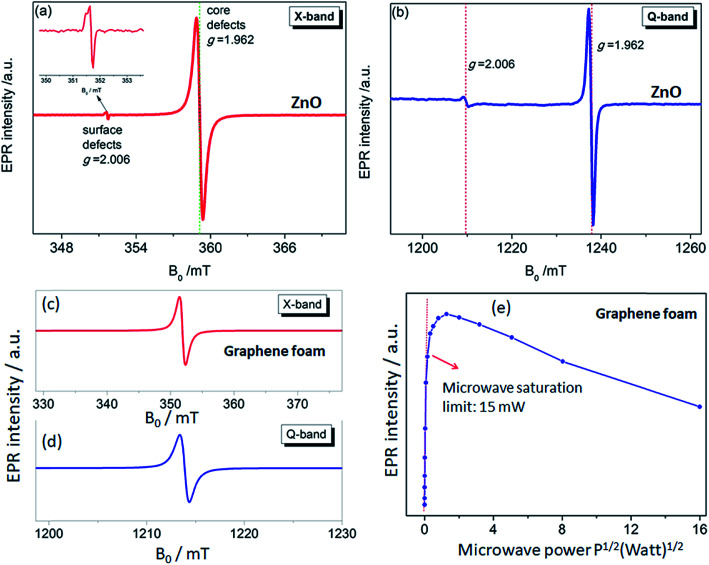
Room temperature continuous wave EPR signals of ZnO nanocrystals measured at the (a) X-band and (b) Q-band. Room temperature continuous wave EPR signals of GF measured at the (c) X-band and (d) Q-band, respectively. (e) The EPR intensity was extracted from the microwave power-dependent spectra in order to understand the saturation behavior.

The use of multifrequency EPR (X- (9.86 GHz) and Q-band (35 GHz)) to detect and assign the defect kinds is a powerful approach. For this purpose, ZnO and GF electrodes were investigated both regarding the X- and Q-bands. The advantage of using multifrequency is the ability to attain a better sensitivity and to resolve anisotropy in the g-factor and the hyperfine structure if they exist. The ZnO electrode used here revealed two distinctive EPR spectra both in the X- and Q-bands, which were reported and explained previously by Erdem *et al.* by the aid of the so-called core–shell model.^11,17^ According to this model, ZnO has paramagnetically active defect centers, namely singly ionized V_O_, V_Zn_, Zn, and O interstitials. The assembly of such defects or defect complexes consisting of such paramagnetic active centers gives two distinct EPR centers, as shown in Fig. 4(a) and (b), at *g* = 1.962 and *g* = 2.006 depending on their location inside the ZnO nanocrystal lattice. In the case the ionized trapped electrons or holes are localized in the core/bulk sides of the crystal, they are more bound and feel a larger spin–orbit coupling effect, causing a large deviation from the *g* ≈ 2 region and yielding a *g*-factor of 1.962. Whereas, if the ionized electrons are located near the surface, they are delocalized and behave like free electrons with a *g*-factor of 2.006 (*g*_free-electron_ = 2.0023). These defect centers also contribute as conduction electrons and increase the conductivity of the ZnO sample enormously. Thus, nanoscale ZnO has different electrical-transport properties than the bulk ZnO counterpart, mainly due to the existence of defect centers. Such electrical-transport properties of defects in ZnO nanocrystals have been presented earlier by the aid of EPR and two point dc electrical results.^11^ We reported that the specific resistivity of ZnO is reduced drastically with decreasing the crystallite size, thus increasing the concentration of surface defects. Detailed EPR analysis of systematically synthesized ZnO nanocrystals *via* the solid-state reaction method has already been reported elsewhere,^13^ and thus further analysis of the EPR on ZnO sample would be redundant here.

The defect structures of GO or rGO,^44^ carbon nanotubes (CNT),^45^ and carbon dots (Cdots)^5^ have been investigated by EPR; however, there is no EPR report on 3D-GF. In Fig. 4(c) and (d), the X- and Q-band EPR results of the GF electrode are presented and a Lorentzian line shape were obtained for both microwave frequencies. Such a single EPR spectrum is expected for carbon-related samples and indicates the defect centers due to dangling bonds and carbon radicals. Such defect centers can be correlated with the Raman data since the disorder in graphene can be described by the intensity of the D-band. Although we did not observe any D-band in the Raman spectra; nevertheless, according to the present EPR and Raman data, the defect contribution to conductivity is consistent and such crystallographic and electronic properties definitely play a key role in the performance of the ultimate energy storage devices, here *superbat*s. Of course, we should admit that the conductivity discussion here is not done by direct measurements but rather in an indirect way *via* EPR and Raman. Besides, it is quite a difficult task to understand the role of defects in GF and ZnO once both electrodes are working synergistically together with the electrolyte in a device. The correlation between the Raman and EPR data is further discussed below.

In order to understand in detail the dependency of the carbon-related defects to the microwave power, we investigated the saturation behavior of the EPR spectra by X-band EPR. In Fig. 4(e), the peak-to-peak intensity of the EPR spectra for the GF signal rise with the increasing microwave power. However, it then deviates and starts damping strongly at 15 mW, indicating a saturation behavior. This observation indicates that we can safely make EPR measurements below 15 mW without saturating the signal intensity. Furthermore, power saturation measurements in EPR spectroscopy give detailed information on the dynamic properties of defect centers. This procedure provides a simple alternative method to obtain spin-lattice (*T*_1_) or spin–spin (*T*_2_) relaxation data when direct-pulse-saturation EPR techniques are not available or are less suitable.^14,46^ In EPR spectroscopy, it is well known that^47^ easy-to-saturate systems have generally long relaxation times, whereas hard-to-saturate systems have short relaxation times. From the microwave power-dependent measurements, it was seen that the GF defect centers were highly steady due to its easy saturation behavior, with an inhomogeneous broadening of the Gaussian line shape. In addition, the number of defect centers can be quantitatively determined by the aid of EPR spectra independent from the microwave frequency. In order to calculate the defect concentration, in other words the carbon concentration, the EPR first-derivative signal is doubly integrated. By comparing the integral of the standard sample (here, MnO powder with 1.495 × 10^18^ spins) and the measured sample (here, GF), the corresponding number of spins can be obtained, and thus the absolute concentration of defect centers. For an accurate determination of the defect concentration quantitatively, the spin-counting procedure has to be carefully considered, as indicated in a well-known text book^48^ and in our previous publications.^11,14,15,41^ According to the spin-counting results, 8.2 × 10^19^ spins per g paramagnetic defects were obtained, which is a relatively high number. Moreover, sp^2^- and sp^3^-hybridizations can be differentiated by their linewidths (Δ*B*) in the EPR spectra.^18,49–51^ The linewidth for sp^3^-hybridized carbon centers, as found in diamond for instance, has been determined as Δ*B*_sp^3^_ < 1 mT, while for graphite-like carbon centers with sp^2^-hybridization, generally a broader Δ*B*_sp^2^_ ≥ 1 mT is observed. The corresponding values of Δ*B* for our GF sample in the X- and Q-band spectra are presented in Fig. 4(c) and (d) and lie around 1.02 mT both in the X- and Q-band spectra, allowing us to experimentally determine the hybridization state of carbon. These results strongly supported the mere presence of sp^2^-bonding. Here, one can extend the discussion to the absence of the D-band and the pronounced existence of a two phonon 2D-band in the Raman results given in Fig. 2. It is well known that graphene has sp^2^ bonds between carbon atoms that are not related with the defect centers, whereas sp^3^ bonds (like diamonds) are strongly related to the defect structures. Actually, the existence of structural defects in sp^2^-hybridized carbon bonds reveals the D-band in the Raman spectra, and thus makes Raman spectroscopy one of the most sensitive methods to investigate defects in sp^2^ carbon materials, here GF. The G-band in Fig. 2 was caused by scattering by Brillouin zone center phonons, whereas the 2D-band (also referred to as G') has been observed when sp^2^ carbons exist, and can be attributed to resonant Raman scattering processes.^52–54^ The line shape and the relative intensity of the 2D-band can be used to determine the number and orientation of graphene layers in few-layered graphene samples (1–5 layers).^52^ Indeed if the graphene is defect-free, the 2D-band is expected to have a high intensity and a sharp lineshape. The present Raman data highly suggest that the G- and 2D-bands are highly sensitive modes to the charge transfer induced in GF, which are due to the electron–phonon-coupling-induced renormalization of the phonon energy. On the other hand, the absence of a D-band suggests that the sample is defect free,^55,56^ while the D-band is caused by the disordered structure in carbonaceous materials, here 3D-GF. However, the defects may not be visible in the Raman spectra simply because of two main reasons. First, EPR-active defect centers, such as carbon dangling bonds and π-conjugated carbon radicals in GF, have a silent mode, A2, which is Raman inactive. Second, their number is below the Raman detection limit if the measurement volume is small. This problem can be overcome by the EPR technique, as its detection limit of approximately 10^11^ spins^57,58^ is extremely low compared to Raman spectroscopy. Moreover, Raman is somewhat an indirect observation of defect centers *via* phonon modes, where the defect centers affect the Raman-active vibrational modes, while EPR is a direct method to detect the defect states. Thus, it is important to note that the two methods have different detection mechanisms and sensitivity levels. Nevertheless, although Raman data do not indicate any defect structure (almost defect-free) by the absence of a D-band, both methods used here indicated sp^2^ bonding for the GF.

**Fig. 5:**
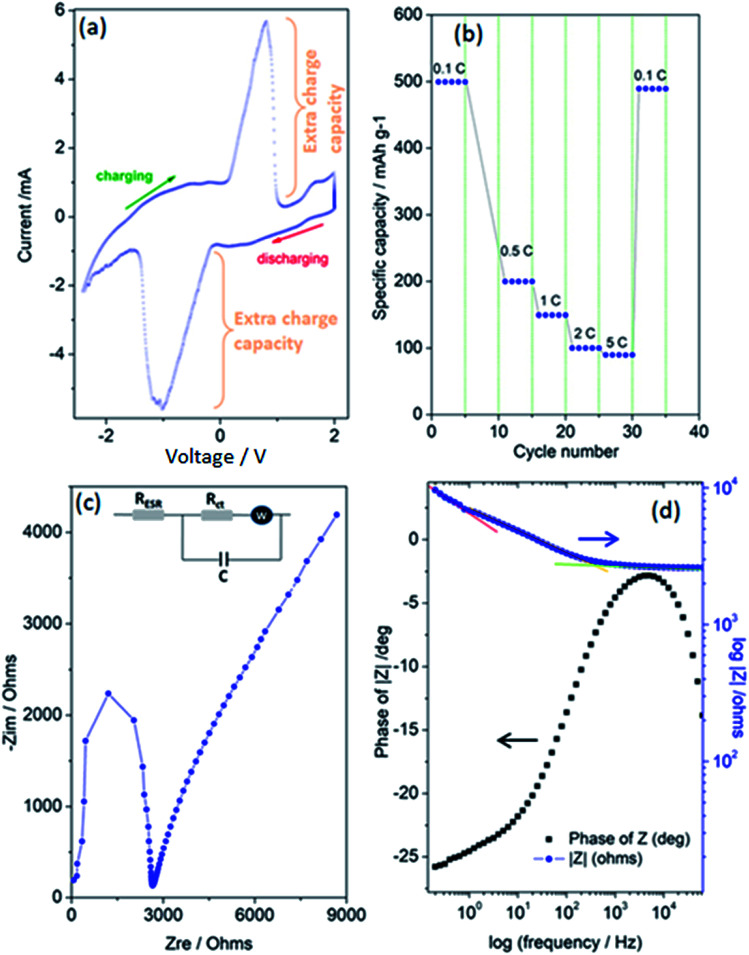
Electrochemical performance results of the *superbat* designed with ZnO nanocrystal and graphene foam electrodes, LiPF_6_ electrolyte, and a glass fiber separator. (a) Cyclic voltammetry (CV), (b) rate performance obtained from the galvanostatic cycle with potential limitation (GCPL) technique. (c) Nyquist plot and (d) Bode plot obtained from electrochemical impedance spectroscopy (EIS).

Finally, Fig. 5(a–d) present the outstanding electrochemical performance results of the *superbat* system produced from the electrode materials described above and then characterized. Collectively, these results show that the assembled *superbat* performed close to perfection in its own frame. In Fig. 5(a), a typical pseudocapacitance CV profile is presented, showing that the shape of the CV is related to the energy storage mechanisms, *i.e.*, rectangular for most EDLCs or quasi-rectangular for pseudocapacitors due to the redox peaks.^59^ It is well known that such Faradaic supercapacitors have surface-confined *I*–*V* curves due to their kinetics. Their mechanism consists of two main parts: a surface redox and intercalation process.^21^ In both mechanisms, during the change in oxidation state and intercalation, unlike batteries, the oxidation state maintains its change continuously without any phase transition. Here, it shows highly reversible redox reactions and in addition presents very large extra charge capacities with a cathodic peak in the charging potential window from 0–1 V with a maximum at 0.79 V, and in the reverse process, an anodic peak in the discharging potential window from 0.12–1.5 V (negative), with a minimum at −1.05 V. Such an extra charge capacity obtained by the oxidation/reduction peak couples increase the system's conductivity as well as energy storage capacity. It is important to note that these peaks inherently exist with capacitive currents without any migrations or any kind of decomposition in electrodes or electrolyte materials. Such a surface-related capacitive process increases the charge-storage capabilities. Of course, it is not easy to distinguish here whether the high current peaks were due to the redox-active ZnO nanocrystal or 3D-GF solely or both. Probably, both electrodes, including their defect structures on the surface, make significant contributions simultaneously; however, they overlap at more or less the same range of voltage window and cannot be resolved here. That's why only one peak per redox is observable. Thus, it is possible to conclude here that the extra charge storage at the interface of the electrolyte and electrode materials, and efficient electron transport due to the increased number of surface defects affect the electrochemical behavior of *superbat*. Moreover, the very large peak-to-peak potential separation and current peaks is a good observation of a high electron-transfer rate and a larger effective surface area for the *superbat* system. Of course, this statement is quite debatable and a few scientists have reported that a large peak-to-peak potential separation is an indication of poor performance due to high resistance in the system due to a low electron-transfer rate. However, for instance, such a large shift in both the cathodic and anodic peaks has been attributed to edge-plane defects in graphite basal plane structures used as electrodes,^60,61^ resulting in an extraordinary good electrochemical performance.

In Fig. 5(b), the rate performances were evaluated from 0.1 to 5C with 5 cycles at each rate. Discharge capacities of 499, 199, 150, 99, and 89 mA h g^−1^ were obtained at 0.1, 0.5, 1, 2, and 5C, respectively. When tuning the current density back to 0.1C, the capacity mostly recovered, having the value of 489 mA h g^−1^. Conventionally, this indicates the good cycling stability of *superbat* at various current densities, indicating a negligible loss of active material due to any dissolution or decomposition of the electrolyte or electrodes or the occurrence of any kind of shuttling during cycling. The superior performance of the *superbat* can be ascribed to the GF's well-developed 3D porous structure and ZnO's nanoscale particle size, hence defect structures. The high surface area micropores of GF can strongly adsorb extra conduction electrons due to the defects, whereas the distribution of defects on the surface of the small-sized ZnO nanocrystals together facilitate rapid mass diffusion and transport to access the redox active sites. Therefore, when utilized as the *superbat* as a whole, the electrodes can rapidly trap electrons due to the defects and prevent their migration to enhance the *superbat* performance. At this point, we have to emphasize that indeed ZnO is a battery-type material and not a pseudocapacitive one. The CV in Fig. 5(a) is typical of a battery, not a pseudocapacitive material, which in normal conditions would give a rectangular shape CV. Actually, there is much confusion in the scientific literature between pseudocapacitance and battery capacity.^62,63^ True pseudocapacitive materials are in general RuO_*x*_ and MnO_*x*_, which provide typical rectangular CVs. If there are strong peaks, as in this case, it is not pseudocapacitance behavior but battery behavior; that's why we named our system as *superbat*, while this system both behaves like a battery and supercapacitor and where the capacity describes both the storage of energy and capacitance. Therefore one may say here that nano-sized ZnO shows an exceptional extraordinary electrochemical performance behavior more than the counterpart bulk ZnO due to its own surface defects.^14^ More than this, two scenarios can be put forward here for the significant electrochemical performance: (i) it is due to lithium intercalation into the ZnO electrode lattice since many literature studies have reported Li-insertion into ZnO nanocrystals or (ii) it is due to defect-assisted Li-intercalation into ZnO nanocrystals. One drawback here can be given as follows: from previous literature, the graphene 3D foam as a supercapacitor electrode itself does not exhibit such redox-like cycling behaviors since it should be limited to surface adsorption processes during charging–discharging. The Nyquist plot and Bode plot of the phase angle and total impedance in Fig. 5(c) and (d) were obtained from the EIS data, respectively. EIS measurements can provide information on circuit components, such as equivalent series resistors, capacitors, diffusion parameters, and charge-transfer resistances that cannot be obtained in more detail and clearly by CV or GCPL techniques. Fig. 5(c) shows a well-defined semi-circle showing a typical Nyquist property followed by a non-Ohmic increase. In the inset of Fig. 5(c), an equivalent Randles circuit of the system is given, which is a common physical model of the electrode–electrolyte interface that is constructed according to some general rules described elsewhere.^64^ According to this circuit, the system consists of the following electrical components: equivalent serial resistance (*R*_ESR_), an active charge-transfer resistance (*R*_ct_), Warburg diffusion element (*W*), and pseudocapacitance (*C*). The EIS analyses revealed that the serial resistance of the *superbat* was 2.65 kΩ, whereas the charge-transfer resistance *R*_ct_ was 8.65 kΩ. Here, the existence of the Warburg impedance element indicates the ready diffusion of the electrolyte ions and the good capacitive behavior as well.^65^*R*_ct_ and pseudocapacitance (*C*) appear in the Randles circuit due to the surface roughness and conductivity difference between the electrodes and electrolyte. In Fig. 5(d), the variation of the impedance as a function of the frequency confirmed the corroboration with the capacitance measurements, while the curve followed the expected Kramers–Kronig relation, which showed an obvious increase in the impedance at lower frequencies.^5,66^ It also showed three significant slopes at different frequency regions for the entire *superbat* system; in other words, for the Randles circuit given in the inset in Fig. 5(c). At the low-frequency region (0.1–0.7 Hz), the slope is quite steep (red line in Fig. 5(d)), due to the less conductive component of the interfaces. At the intermediate-frequency region (0.7–700 Hz), the slope is somehow less (orange line in Fig. 5(d)) than that in the lower frequency range, indicating that both the capacitive and resistive components of both electrodes at the interfaces are active and compete with each other. This more resistive region actually dominates the system through its wide range of frequency dependency. Finally, at the high-frequency region (>1000 Hz), the slope is almost flat (green line in Fig. 5(d)) meaning that *superbat* is highly capacitive at this range, where the capacitance *C* controls the overall system. Such a frequency dependency of the impedance can be attributed to the remarkable electrochemical performance of both electrodes, giving a high specific capacitance and good rate capability in the *superbat* system. The explanation for this is indeed quite simple: the high conductivity of graphene foam assists electron transfer and enables the easy transport of electrolyte ions (PF_6_^−^, Li^+^) to the electrode surface. Moreover, the contact between the surface defects in both electrodes and the electrolyte is increased during the electrochemical reaction. Thus, defect structures play an important role in improving the electrochemical performance of *superbat* and prove that these electrode materials (ZnO and 3D-GF) can be effectively used for the improvement of the performance of supercapacitors. The maximum point of the Z-phase is also shown in Fig. 5(d) as −2.7° (near 0°) at 4.42 kHz, which corresponds to an almost linear behavior (on the Bode plot of frequency *versus* impedance) with a slope of ∼1. Finally, *superbat* exhibited a specific capacity of 498 mA h g^−1^ at 0.1C and showed excellent rate capability. More importantly, the pseudocapacitive *superbat* produced by the synergetic effects of ZnO and 3D-GF electrodes could be operated in a two-electrode system in the 0–2 V voltage region and gave a high specific capacitance of 448 F g^−1^, which delivered a maximum energy density of 35 W h kg^−1^ at a maximum power density of 270 W kg^−1^.

## 4. Conclusions

A special design called *superbat* was constructed and the role of defect structures in ZnO and GF were investigated for the improvement of the electrochemical performance. The synergetic effects of both electrodes and the electrolyte revealed high values for the energy density and power density. Here we studied the aforementioned electrode and electrolyte materials in depth and found out that the intrinsic defect states play an essential role in the electrical and electronic properties, and showed that the detailed investigations of such defects are generally underestimated so far in the literature. Various state-of-art characterization techniques, such as electron paramagnetic resonance, and Raman and impedance spectroscopy, were undertaken in order to understand the origin of the performance of the hybrid capacitor in more detail. In particular, we obtained a high capacitance value (*C* = 448 F g^−1^), which was exceptionally related not only to the quality of synthesis but also to the choice of electrode and electrolyte materials. Moreover, not only did each component used in the construction of the hybrid supercapacitor play a key role in achieving the high capacitance value, but also the smart supercapacitor design allowed extensive control of the mounting of each component. This contribution contains interesting new science covering several different aspects related to nanomaterials and defect-related science in such materials. It is therefore hopefully interesting for materials scientists and chemists as well as engineers working in the field of hybrid capacitors. Moreover, this work may be of high interest to a variety of researchers working on the development of electrode materials for efficient supercapacitors.

## Conflicts of interest

There are no conflicts to declare.

## Supplementary Material
